# Shwachman-Bodian-Diamond syndrome protein desensitizes breast cancer cells to apoptosis in stiff matrices by repressing the caspase 8-mediated pathway

**DOI:** 10.1080/19768354.2019.1666030

**Published:** 2019-09-20

**Authors:** Jieun Lee, Panseon Ko, Eunae You, Jangho Jeong, Seula Keum, Jaegu Kim, Mizanur Rahman, Dong Ho Lee, Sangmyung Rhee

**Affiliations:** Department of Life Science, College of Natural Sciences, Chung-Ang University, Seoul, Republic of Korea

**Keywords:** SBDS, breast cancer, ECM stiffness, apoptosis, caspase

## Abstract

Certain cancer types, including breast cancer, are accompanied with stiffening of the surrounding extracellular matrix (ECM). Previous studies suggest that this stiffened matrix influences cancer cell progression, such as proliferation and invasion, both biochemically and mechanically. However, the contribution of ECM stiffness to cellular response to diverse stresses, which most cancer cells are exposed to, has not been elucidated. In this study, we demonstrate that expression of the Shwachman-Bodian-Diamond syndrome protein (SDBS) in a stiff matrix protects cells from apoptosis induced by environmental stress, including anticancer drugs. Cells cultured on stiff matrices were less apoptotic process induced by serum depletion than those cultured on the soft matrix. Interestingly, knockdown (KD) of *SDBS* among the apoptosis-related genes significantly increased apoptosis induced by serum depletion in cells cultured in a stiff matrix. Apoptosis of *SDBS* KD cells in a stiff matrix was significantly inhibited by the caspase 8 inhibitor, indicating that activation of the caspase 8 pathway by *SDBS* KD is critical for cancer cell apoptosis in stiff matrices. Additionally, we also found that downregulation of *SDBS* also effectively increased cell death induced by anticancer drugs, including paclitaxel, cisplatin, and eribulin. Taken together, our findings suggest that inhibition of *SDBS* enhances effective chemotherapy of malignant breast cancer cells in stiff ECM environments.

## 1. Introduction

Progression of solid tumor, as in breast cancer, is accompanied with stiffening of the extracellular matrix (ECM) (Lampi and Reinhart-King 2018). Increased ECM rigidity and the consequential mechanical cue as cancer progresses results in different cancer cell responses to their environment, in terms of morphology and gene expression (Provenzano et al. 2009). Previous studies indicated that matrix stiffing is particularly involved in gene expression related to proliferation and invasion of cancer cells. For example, upregulation of spindle pole body component 25 homolog (SPC25) gene in stiff matrices is required for proliferation of H1299 lung adenocarcinoma cells by increasing the accessibility of the chromosome alignment in metaphase (Jeong et al. 2018). However, although cancer cells are exposed to numerous stresses, such as oxidative and metabolic stress, the effect of ECM rigidity on cellular stress response is not fully understood (Visconti and Grieco 2009; Li et al. 2011).

Apoptosis is programmed cell death and one of the responses to cellular stress (Herr and Debatin 2001). This highly regulated process is activated by cellular signals, such as DNA damage or growth factor deprivation (Araki et al. 1990; Roos and Kaina 2013). Apoptosis is initiated via two major pathways, mitochondrial and death receptor-mediated pathways, which are also known as intrinsic and extrinsic pathways. The intrinsic pathway is induced by an intracellular signal (Haupt et al. 2003), which leads to the release of mitochondrial cytochrome C into cytosol, then, the cytochrome C triggers caspase 9/3 cascades (Ow et al. 2008). The extrinsic pathway begins with the reception of the extracellular signals by death receptors, including Fas, inducing apoptosis through caspase 8/3 cascades (Wajant 2002). Intriguingly, cancer cells are renowned for evading apoptosis via several mechanisms, therefore, apoptosis regulation is considered to be crucial for cancer therapy (Fernald and Kurokawa 2013).

The Shwachman-Bodian-Diamond syndrome protein (SBDS) was first identified as a protein, which when mutated causes the Shwachman-Diamond syndrome (SDS), an inherited disorder characterized by bone marrow failure (Woloszynek et al. 2004; Kawakami et al. 2005). The precise function of SBDS is unknown, however its contribution to biogenesis, maturation and translational activation of ribosome has been suggested (Ganapathi et al. 2007; Menne et al. 2007). Furthermore, SBDS is now known to be involved in protection of cells from apoptosis via the Fas-mediated pathway. SBDS-deficient HeLa cells underwent both the accumulation of Fas, a member of the tumor necrosis factor receptor family, at the plasma membrane and the acceleration of apoptosis (Rujkijyanont et al. 2008; Watanabe et al. 2009; Ambekar et al. 2010). Mutation of the SBDS gene is known to increase the risk of leukemia, but its association with solid tumors, including breast cancer, remains unknown (Majeed et al. 2005).

In this study, we found that cellular apoptosis of MDA-MB-231 cells in response to specific stress, such as serum starvation, decreases in stiff substrates. Furthermore, SBDS was identified as a promising regulator through screening on both stiffness-dependent mRNA expression of apoptosis regulators and hazard ratio of breast cancer patients. *SBDS* silencing by siRNA promoted apoptosis exclusively in the stiff substrates, but not in the soft substrates, suggesting that SBDS regulates stiffness-dependent apoptosis. We also confirmed that the caspase 8-mediated apoptosis pathway is involved in this process. Furthermore, genetic knockdown of *SBDS* sensitized breast cancer cells to anticancer drugs, including paclitaxel, cisplatin, and eribulin. Therefore, our results suggest that, in stiff substrates, mRNA expression of *SBDS* is upregulated and it blocks the caspase 8-mediated pathway, resulting in decreased apoptosis.

## 2. Materials and methods

### 2.1. Cell culture

Human breast cancer MDA-MB-231 cell line was purchased from Korea Cell Line Bank (Seoul, Korea). The MDA-MB-231 cells were maintained in Roswell Park Memorial Institute 1640 medium (RPMI 1640; Gibco-BRL, Grans Island, NY, USA) supplemented with 10% fetal bovine serum (FBS; YoungIn Frontier, Seoul, Korea), 100 units/ml penicillin and 100 μg/ml streptomycin (Welgene, Seoul, Korea). Cells were incubated at 37°C with 5% CO_2_.

### 2.2. Preparation of polyacrylamide gel (PAG) matrices

To produce PAG matrices with the desired elasticity, acrylamide and bis-acrylamide were blended in appropriate proportions. The mixture was placed on a 25 mm coverslip activated with 2% 3-aminopropyltriethoxysilane (Sigma-Aldrich, Taufkirchen, Germany). Then, the matrices were coated with 0.5 mg/ml sulfosuccinimidyl 6-(4ʹ-azido-2ʹnitrophenyl amino) hexanoate (ProteoChem, CO, USA) and incubated with 50 μg/ml collagen at 4°C overnight. The rigidity of the PAG matrices was defined by Atomic Force Microscopy (NX10, Park systems Corp., Suwon, Korea).

### 2.3. Cell viability assay

Live/Dead^TM^ Viability/Cytotoxicity Kit (Invitrogen, CA, USA) was used to evaluate cell viability. MDA-MB-231 cells seeded on 12 mm coverslips were incubated with 2 μM Calcein AM and 4 μM EthD-1 at room temperature for 30 min. Then, the coverslips were mounted on slide glasses and observed under a fluorescent microscope. Cell viability was analyzed by assessing the percentage of red fluorescent cells in all cells. To determine the viability of cells which were treated with anticancer drugs, the 3-(4,5-dimethylthiazol-2-yl)-2.5-diphenyltetrazolium bromide assay (MTT) was performed. Both SBDS-knockdown and control cells were plated in 96-well plates in triplicate wells. Absorbance was measured at 570 nm using a microplate reader (Bio-Tek Instrument, Inc., Winooski, VT, USA).

### 2.4. Apoptosis assay

To measure DNA content, cells were fixed using 70% ethanol and stained with propidium iodide (PI). Utilizing BD accuri C6 Plus (BD Biosciences, San Jose, CA, USA), the sub-G1 population, representing apoptotic cells, was evaluated. For another apoptosis analysis, ApoScreen Annexin V Apoptosis Kit-FITC (Southern Biotech, Birmingham, AL, USA) was used. Cells were stained with both Annexin V-FITC and PI and then, analyzed using a flow cytometer. Total apoptotic cells were determined as the sum of Annexin V^+^/PI^-^ (early apoptotic) cells and Annexin V^+^/PI^+^ (late apoptotic) cells.

### 2.5. RNA isolation and quantitative RT–PCR

Total RNA was extracted using RNAiso Plus reagent (TaKaRa, Tokyo, Japan) according to the manufacturer’s protocol. The complementary DNA (cDNA) was synthesized using PrimeScript^TM^ Reverse Transcriptase and ribonuclease inhibitor (TaKaRa). Quantitative RT–PCR (qRT-PCR) was carried out with TB Green^TM^ Premix Ex Taq^TM^ II (TaKaRa) utilizing Quant Studio 3 Real-Time PCR System (Applied Biosystems, CA, USA). The data were analyzed according to the 2^-ΔΔCt^ method and normalized with the Ct value of glyceraldehyde-3-phosphate dehydrogenase (GAPDH).

### 2.6. siRNA-mediated knockdown

siRNA oligos targeting SBDS (siRNA #1: 5ʹ-AAGCUUGGAUGAUGUUCCUGAUUUU-3ʹ, siRNA #2: 5ʹ-AACAUGCUGCCAUAACUUAGAU-3ʹ) and control mock siRNA were used. The siRNAs were transfected using Oligofectamine transfection reagent (Invitrogen) and incubated 24 h.

### 2.7. Statistical analysis

Differences between the groups were analyzed with the Student’s *t*-test via GraphPad PRISM (Graphpad Software, CA, USA) for their statistical significance. All data were obtained from at least three independent experiments and presented as average ± standard error of the mean (SEM). Results with *p*-values less than 0.05 were considered to be statistically significant.

## 3. Results

### 3.1. MDA-MB-231 cells are more resistant to apoptosis in stiff environments

**Figure 1.**
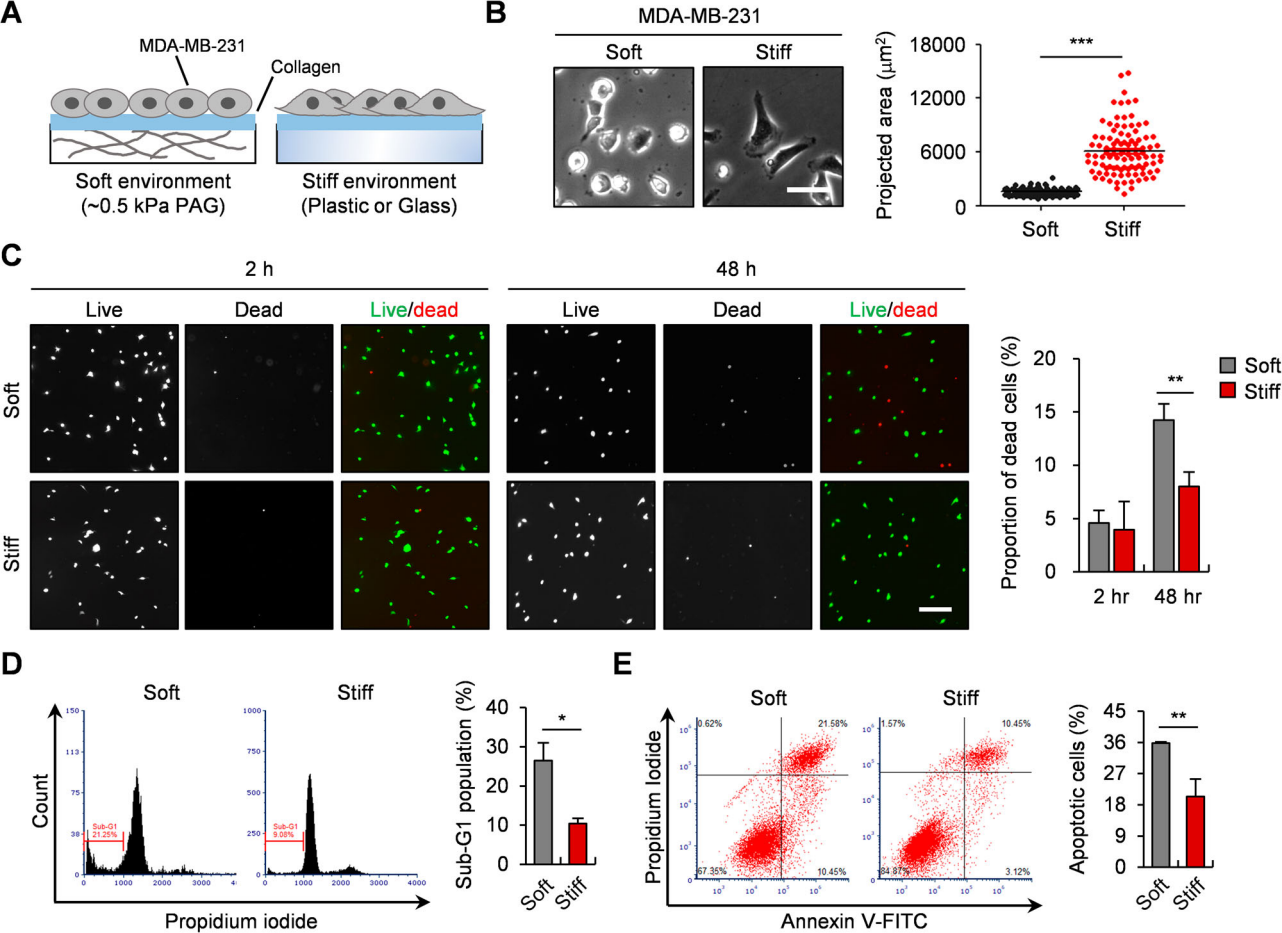
Apoptosis of MDA-MB-231 cells is decreased in a stiff environment. (A) Schematic diagram of the experimental procedure. PAG matrices (∼0.5 kPa), plastic dishes and glass coverslips were utilized to imitate soft and stiff environment, and were coated with collagen (50 μg/ml) before seeding MDA-MB-231 cells. (B) Representative images and the projected area (*n* = 100) of MDA-MB-231 cells in soft or stiff environments. Scale bar, 50 μm. (C) Live/dead staining images of MDA-MB-231 cells. Cells were incubated without serum on PAG matrices (∼0.5 kPa) or glass coverslips for 2–48 h. Dead cell population was determined by counting the live (green fluorescent) cells and dead (red fluorescent) cells. Scale bar, 100 μm. (D) Cell cycle profiles of MDA-MB-231 cells on PAG matrices (∼0.5 kPa) or plastic dishes after PI staining. Population of cells in sub-G1 phase, representing apoptotic cells, is indicated in percentages. (E) Total apoptotic cells on PAG matrices (∼0.5 kPa) or plastic dishes were determined by Annexin V/PI staining. Proportion of apoptotic cells is indicated in percentages. Data represent the mean ± S.E.M. *p < 0.05; **p < 0.01; ***p < 0.001.

To evaluate matrix stiffness-dependent apoptosis of breast cancer cells, MDA-MB-231 cells were cultured on soft or stiff substrates, under cellular stress induced by serum starvation (Figure 1(A)). The breast cancer cells were more spread out on the stiff substrates than the soft substrates and, therefore, the projected area of the cells increased on the substrates (Figure 1(B)). To measure cell death depending on substrate stiffness, the proportion of dead cells was first determined by live/dead staining assay. After 48 h, the proportion of dead cells on the soft substrates had increased more than two times those on the stiff substrates (Figure 1(C)). Then, to figure out whether this cell death was induced by apoptosis, the apoptosis rate was evaluated by both PI staining and Annexin V/PI double staining. The percentage of cells in the sub-G1 phase, which represent apoptotic cells, decreased by ∼16% in the stiff substrates than in the soft substrates (Figure 1(D)). The proportion of apoptotic cells (Annexin V^+^/PI^-^ and Annexin V^+^/PI^+^) also decreased by ∼15% in the stiff substrates compared to the soft substrates (Figure 1(E)). Together, these results indicate that apoptosis of breast cancer cells is downregulated in stiff substrates and suggest the possibility of a substrate stiffness-dependent mechanism which regulates cellular apoptosis.

### 3.2. Transcript level of SBDS is increased in cancerous environment

**Figure 2.**
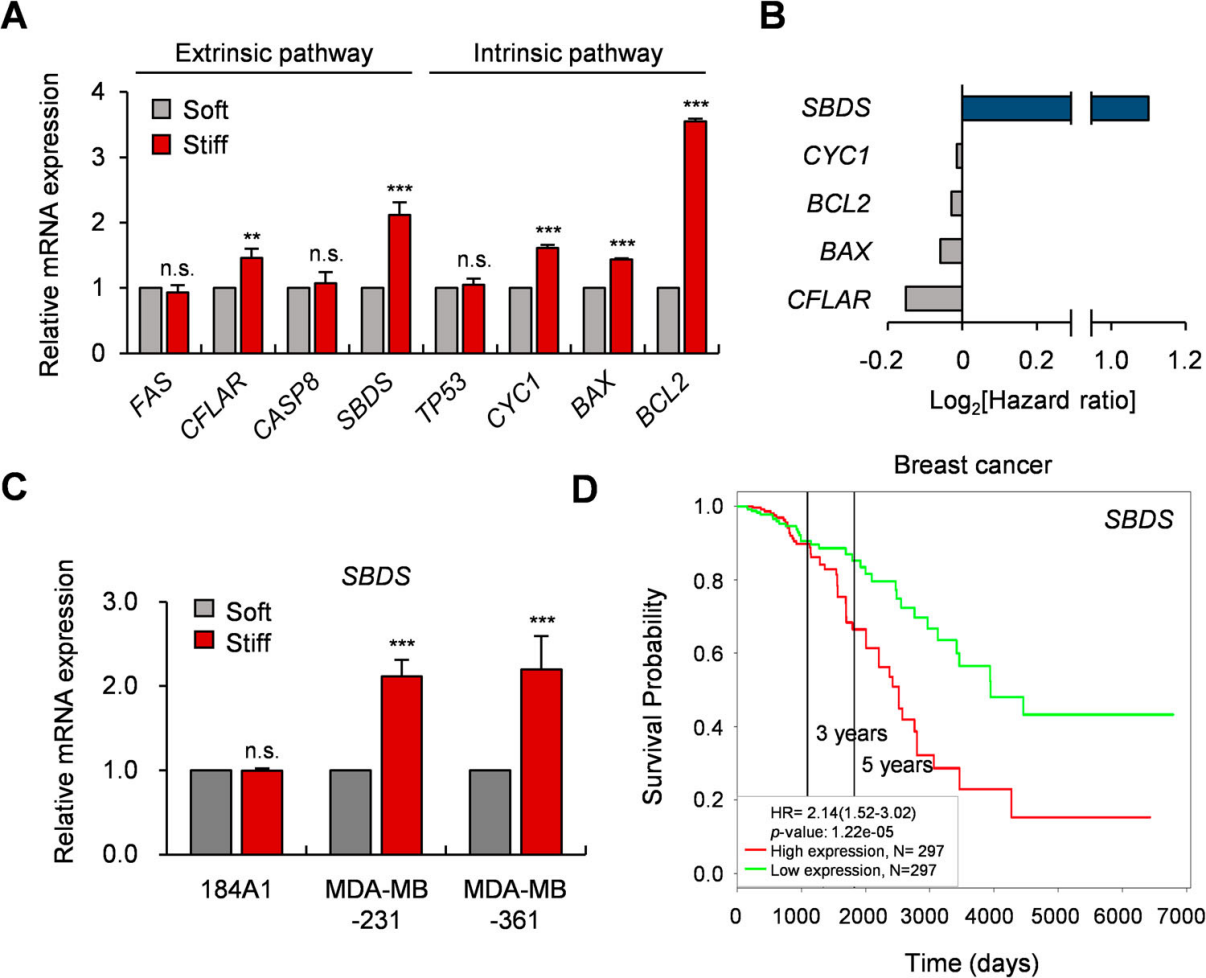
*SBDS* expression is upregulated in the stiff substrates. (A) The relative mRNA levels of apoptosis-related genes in the soft or stiff substrates. The transcript expression of the extrinsic and intrinsic pathway-related genes were inspected by qRT-PCR. (B) The hazard ratio of five genes which were more significantly upregulated in the stiff substrates than in the soft substrate. The blue-colored bar indicates the gene with the highest hazard ratio among the five genes. The hazard ratio was determined by TCGA-BRCA dataset. (C) Substrate stiffness-dependent mRNA expression of *SBDS* in breast normal and cancer cell lines. (D) Survival analysis in relation to *SBDS* expression of TCGA-BRCA dataset available on PROGgene V2 website (http://genomics.jefferson.edu/proggene/). Data represent the mean ± S.E.M. n.s., *p ≥ 0.05; **p < 0.01; ***p < 0.001.

The apoptotic pathway is known to be initiated through a mitochondria-mediated intrinsic pathway and death receptor (Fas, TNF-R)-mediated extrinsic pathway (Ichim and Tait 2016). To identify the key molecule that regulates stiffness-dependent apoptosis, the transcript levels of apoptosis-related genes (*FAS*, *CFLAR*, *CASP8*, *SBDS*, *TP53*, *CYC1*, *BAX* and *BCL2*) depending on substrate rigidity were examined. Among these, the expression of five genes (*CFLAR*, *SBDS*, *CYC1*, *BAX* and *BCL2*) were significantly upregulated in the stiff substrates and expression level of *SDBS* and *BLC2* genes as negative regulators are often relatively high than others (Figure 2(A)). The hazard ratio of these genes was also investigated based on a database of 297 breast cancer (BRCA) patients (https://portal.gdc.cancer.gov/projects/TCGA-BRCA), and that of *SBDS* was the highest of the five genes (Figure 2(B)). To confirm whether mRNA expression of *SBDS* increased in other breast cell lines, substrate stiffness-dependent expression of *SBDS* was examined in a normal breast cell line, 184A1, and two breast cancer cell lines, MDA-MB-231 and MDA-MB-361. Interestingly, the *SBDS* transcript level of cells in the stiff substrates was exclusively upregulated in two cancer cell lines, but not in the normal cell line (Figure 2(C)). Increase in *SBDS* expression was also associated with poor prognosis for BRCA patients (Figure 2(D)). Therefore, these results suggest that the SBDS is likely to regulate substrate stiffness-dependent apoptosis.

### 3.3. Knockdown of SBDS enhances apoptosis in breast cancer cells

**Figure 3.**
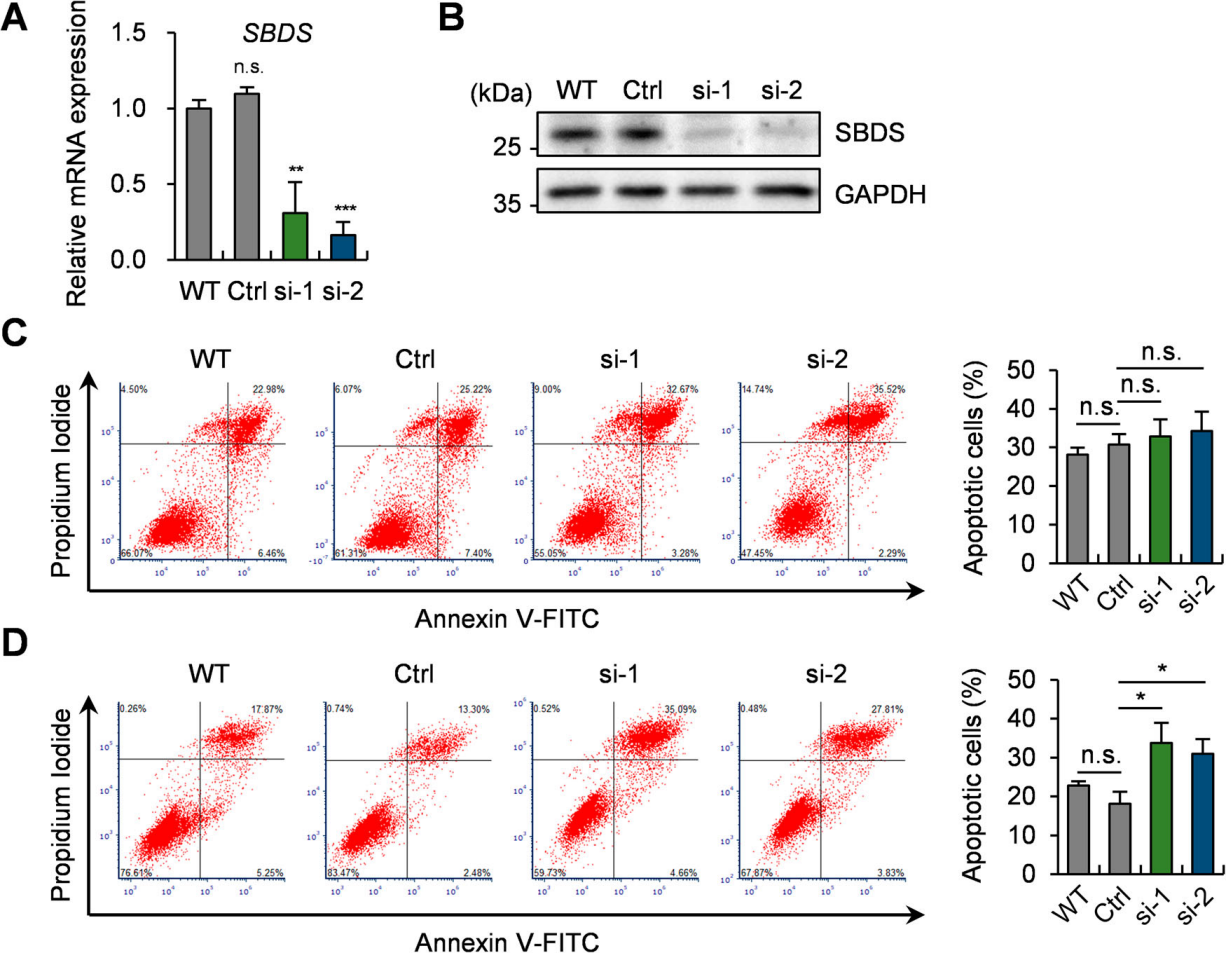
*SBDS* silencing promotes substrate stiffness-dependent apoptosis in breast cancer cells. (A) mRNA expression of *SBDS* in wild-type, control siRNA-transfected and two *SBDS* siRNA-transfected MDA-MB-231 cells. Genetic knockdown of *SBDS* was validated by qRT-PCR. (B) Western blot analysis of SBDS protein expression and GAPDH. (C) Annexin V/PI staining assay of cells in soft substrates. Cells were transfected with siRNA for 24 h and incubated without serum on PAG matrices (∼0.5 kPa) for 48 h prior to the apoptosis assay. (D) Annexin V/PI staining assay of cells in stiff substrates. Experimental procedure was same with (C). Data represent the mean ± S.E.M. n.s., *p* ≥ 0.05; *p < 0.05; **p < 0.01; ***p < 0.001.

To study the function of *SBDS,* with upregulated mRNA expression in breast cancer cells on stiff substrates, genetic knockdown of *SBDS* was achieved with small-interfering RNA (siRNA). As shown in Figure 3(A), two different siRNAs targeting *SBDS* successfully silenced the mRNA level of *SBDS* by ∼70–84%. Its downregulation was also confirmed at the protein level by western blot analysis (Figure 3(B)). To determine whether SBDS is involved in substrate stiffness-dependent apoptosis regulation, the effect of *SBDS* siRNA on apoptosis was evaluated by Annexin V/PI double staining in both soft and stiff substrates. In the soft substrates, the apoptosis rates of wild-type, control siRNA-transfected cells and two *SBDS* siRNA-transfected cells did not have any significant differences (Figure 3(C)). On the contrary, the apoptosis rates of two knockdown cells in the stiff substrates increased about 16% and 13%, respectively (Figure 3(D)). In addition to Figure 2(C), these results indicate that the downregulation of *SBDS* increases apoptosis exclusively in stiff substrates in which mRNA expression of *SBDS* is relatively high. Thus, upregulation of *SBDS* in a stiff matrix plays a role in cellular resistance to apoptosis induced by environmental stress.

### 3.4. SBDS mediates stiffness-dependent apoptosis through caspase 8-related pathway

**Figure 4.**
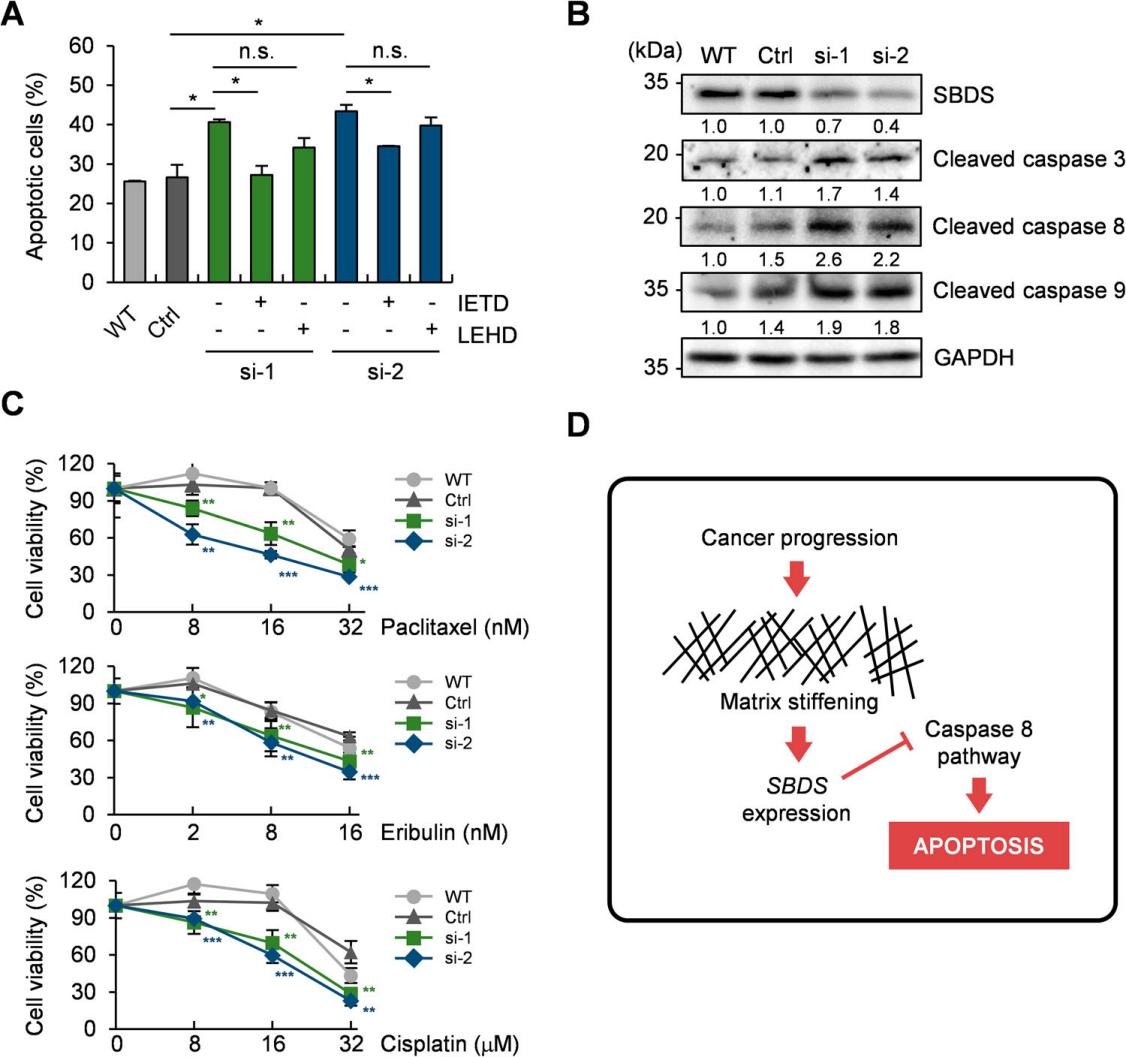
*SBDS* deficiency activates the caspase 8-mediated apoptosis pathway. (A) Annexin V/PI staining assay of wild-type, control siRNA-transfected and two *SBDS* siRNA-transfected cells treated with Z-IETD-FMK or Z-LEHD-FMK in stiff substrates. Cells were cultured in serum-depleted conditions with Z-IETD-FMK (5 μM) or Z-LEHD-FMK (5 μM) for 48 h. (B) Western blot analysis of wild-type, control and two *SBDS* siRNA-transfected cells using antibodies against cleaved caspases 3, 8 and 9, and GAPDH. Cells were transfected with siRNA and incubated for 72 h. (C) Cell viability assay of wild-type, control siRNA-transfected and two SBDS siRNA-transfected cells that were treated with paclitaxel, cisplatin or eribulin. Cells were treated with these drugs at the indicated concentration for 5 days. (D) A schematic diagram illustrating the potential mechanism of SBDS-mediated regulation of substrate rigidity-dependent apoptosis. Data represent the mean ± S.E.M. n.s., *p* ≥ 0.05; *p < 0.05; **p < 0.01; ***p < 0.001.

It has been reported that the extrinsic and intrinsic apoptotic pathways proceed via caspase 8 and 9, respectively, and converge to activate caspase 3 (Li and Yuan 2008). We sought to confirm, using caspase 8 or 9 inhibitors (Z-IETD-FMK or Z-LEHD-FMK), which pathway SBDS inhibition induced or activated. In a similar manner as described in Figure 3(C), the apoptosis rates of two SBDS-knockdown cells were ∼40–43% more than those for control cells. Treatment of *SBDS*-knockdown cells with caspase 8, not caspase 9, inhibitors have resulted in a significant decrease in apoptosis, by approximately 9–13% (Figure 4(A)). These results imply that increased apoptosis due to *SDBS* knockdown is likely mediated by the caspase 8 pathway.

To further confirm whether SBDS inhibits the caspase 8 pathway, we examined the level of activated forms of caspases. The cleavage of caspase 3, a common downstream regulator of both caspase 8 and 9 pathways, increased by *SBDS* knockdown. The activation of caspase 8 was slightly greater than that of caspase 9 in *SBDS*-downregulated cells (Figure 4(B)), indicating that the upregulation of SBDS in a stiff matrix might have a repressive role for cellular apoptosis by inhibiting caspase 8-mediated pathways.

Additionally, we hypothesized that the caspase 8 pathway could affect apoptosis in the soft substrates. Therefore, we examined the death rate of caspase 8 or 9 inhibitor-treated cells in the soft substrates using live/dead staining assay. After 48 h, the proportion of dead cells increased by ∼20% in control cells, whereas the rate of caspase 8 inhibitor-treated cells increased only by ∼10%. Interestingly, the increment in the rate of caspase 9 inhibitor-treated cells was similar to the control suggesting that the caspase 8 pathway is critical to substrate stiffness-dependent apoptosis (Supplementary Figure 1).

### 3.5. SBDS restrains anticancer drug-induced apoptosis

Previous studies revealed that as soon as the surrounding substrates were stiffened by cancer progression, resistance to anticancer drugs increased (Nguyen et al. 2014). For instance, in former studies, mouse mammary carcinoma (MMC) cells and human breast adenocarcinoma (MDA-MB-231) cells showed increased chemoresistance to Doxorubicin, a genotoxic reagent, in the rigid substrates (Chang et al. 2016; Joyce et al. 2018). Therefore, we assumed that the upregulation of apoptosis through *SBDS* knockdown could facilitate the drug accessibility to the cancer cells in the stiff substrates. To prove this, we examined the sensitivity of SBDS-depleted cells in the stiff substrates to paclitaxel, eribulin, and cisplatin, which are antimitotic or genotoxic reagents. As shown in Figure 4(C), *SBDS* KD cells induced effective cell death in the presence of low concentrations of anticancer drugs. Therefore, these results suggest that SBDS inhibition would likely improve the effectiveness of anti-cancer drugs in treating progressive malignant cancer cells localized in aggressive cancer microenvironments, such as stiff ECM.

## 4. Discussion

Increase in ECM rigidity is one of the major features of cancer microenvironments in solid tumors. The rigid ECM results in diverse cellular signaling of cancer cells, including cell proliferation and survival through activated mechanical sensors (Haage and Schneider 2014; Navab et al. 2016). Recently, we and other researchers reported that the expression of numerous genes in cells were altered by matrix stiffening (Ko et al. 2016; Jeong et al. 2018). Genes expressed in the stiff ECM are particularly involved in cell proliferation and survival. However, there are few studies on genes associated with cancer cell apoptosis among the genes regulated by matrix stiffening. Hence, we attempted to identify genes regulating ECM rigidity-dependent apoptosis by examining the transcript levels and hazard ratios in breast cancer cells. Although SBDS has originally identified as a regulator for ribosome biogenesis (Ganapathi et al. 2007), knockdown of SDBS has been reported to increase apoptosis in HeLa cells (Rujkijyanont et al. 2008). In agreement with this finding, *SBDS* knockdown using siRNA, in this study, upregulated apoptosis in the stiff substrates, not in the soft substrates. In addition, SBDS was negatively correlated to the survival of breast cancer patients, implying that *SBDS* is a key regulator of stiffness-dependent apoptosis in breast cancer cells.

Apoptosis proceeds via mitochondrial intrinsic pathway or death receptor-mediated extrinsic pathway (Elmore 2007). The intrinsic and the extrinsic pathways involve caspases 9 and 8, respectively, which converge into caspase 3 (Hongmei 2012). When SBDS is silenced, the Fas receptor is accumulated in the plasma membrane (Watanabe et al. 2009). Our data also revealed that the caspase 8 inhibitor effectively blocked apoptosis which was caused by *SBDS* silencing. In addition, *SBDS* knockdown activated caspase 8, indicating that *SBDS* restrained apoptosis by inhibition of the caspase 8 pathway. Once caspase 8 was activated, it induced the truncation of BID (BH3 interacting domain death agonist) (Li et al. 1998; Kantari and Walczak 2011), which in turn, activates the caspase 9 pathway. Thus, in our results, caspase 9 was also slightly activated in *SBDS*-silenced cells. Nevertheless, it is clear that *SBDS* represses the caspase 8 pathway to block apoptosis.

Resistance to anticancer drugs is a major issue in cancer treatment (Gottesman 2002). Intriguingly, it has been reported that increased ECM stiffness confers anticancer drug resistance (Sharma et al. 2014; Rice et al. 2017). Our data suggest that *SBDS* silencing desensitizes cancer cells in the stiff substrates to anticancer drugs, such as paclitaxel, eribulin and cisplatin. In this regard, SBDS could be a potential prognostic marker and therapeutic target in breast cancer patients.

## Supplementary Material

Supplemental Material

## References

[CIT0001] Ambekar C, Das B, Yeger H, Dror Y. 2010 SBDS-deficiency results in deregulation of reactive oxygen species leading to increased cell death and decreased cell growth. Pediatr Blood Cancer. 55(6):1138–1144. doi: 10.1002/pbc.22700 20979173

[CIT0002] Araki S, Shimada Y, Kaji K, Hayashi H. 1990 Apoptosis of vascular endothelial cells by fibroblast growth factor deprivation. Biochem Biophys Res Commun. 168(3):1194–1200. doi: 10.1016/0006-291X(90)91155-L 2346483

[CIT0003] Chang F-C, Tsao C-T, Lin A, Zhang M, Levengood S, Zhang M. 2016 PEG-Chitosan hydrogel with tunable stiffness for study of drug response of breast cancer cells. Polymers (Basel). 8(4):112. doi: 10.3390/polym8040112 27595012 PMC5004991

[CIT0004] Elmore S. 2007 Apoptosis: a review of programmed cell death. Toxicol Pathol. 35(4):495–516. doi: 10.1080/01926230701320337 17562483 PMC2117903

[CIT0005] Fernald K, Kurokawa M. 2013 Evading apoptosis in cancer. Trends Cell Biol. 23(12):620–633. doi: 10.1016/j.tcb.2013.07.006 23958396 PMC4091735

[CIT0006] Ganapathi KA, Austin KM, Lee C-S, Dias A, Malsch MM, Reed R, Shimamura A. 2007 The human Shwachman-Diamond syndrome protein, SBDS, associates with ribosomal RNA. Blood. 110(5):1458–1465. doi: 10.1182/blood-2007-02-075184 17475909 PMC1975835

[CIT0007] Gottesman MM. 2002 Mechanisms of cancer drug resistance. Annu Rev Med. 53(1):615–627. doi: 10.1146/annurev.med.53.082901.103929 11818492

[CIT0008] Haage A, Schneider IC. 2014 Cellular contractility and extracellular matrix stiffness regulate matrix metalloproteinase activity in pancreatic cancer cells. FASEB J. 28(8):3589–3599. doi: 10.1096/fj.13-245613 24784579

[CIT0009] Haupt S, Berger M, Goldberg Z, Haupt Y. 2003 Apoptosis-the p53 network. J Cell Sci. 116(20):4077–4085. doi: 10.1242/jcs.00739 12972501

[CIT0010] Herr I, Debatin K-M. 2001 Cellular stress response and apoptosis in cancer therapy. Blood. 98(9):2603–2614. doi: 10.1182/blood.V98.9.2603 11675328

[CIT0011] Hongmei Z. 2012 Extrinsic and intrinsic apoptosis signal pathway review. Apoptosis and Medicine. IntechOpen.

[CIT0012] Ichim G, Tait SW. 2016 A fate worse than death: apoptosis as an oncogenic process. Nat Rev Cancer. 16(8):539. doi: 10.1038/nrc.2016.58 27364482

[CIT0013] Jeong J, Keum S, Kim D, You E, Ko P, Lee J, Kim J, Kim J-W, Rhee S. 2018 Spindle pole body component 25 homolog expressed by ECM stiffening is required for lung cancer cell proliferation. Biochem Biophys Res Commun. 500(4):937–943. doi: 10.1016/j.bbrc.2018.04.205 29709477

[CIT0014] Joyce MH, Lu C, James ER, Hegab R, Allen SC, Suggs LJ, Brock A. 2018 Phenotypic basis for matrix stiffness-dependent chemoresistance of breast cancer cells to doxorubicin. Front Oncol. 8. doi: 10.3389/fonc.2018.00337 PMC6134055 30234012

[CIT0015] Kantari C, Walczak H. 2011 Caspase-8 and bid: caught in the act between death receptors and mitochondria. Biochimica et Biophysica Acta (BBA)-Molecular Cell Research. 1813(4):558–563. doi: 10.1016/j.bbamcr.2011.01.026 21295084

[CIT0016] Kawakami T, Mitsui T, Kanai M, Shirahata E, Sendo D, Kanno M, Noro M, Endoh M, Hama A, Tono C. 2005 Genetic analysis of Shwachman-Diamond syndrome: phenotypic heterogeneity in patients carrying identical SBDS mutations. Tohoku J Exp Med. 206(3):253–259. doi: 10.1620/tjem.206.253 15942154

[CIT0017] Ko P, Kim D, You E, Jung J, Oh S, Kim J, Lee KH, Rhee S. 2016 Extracellular matrix rigidity-dependent Sphingosine-1-phosphate Secretion regulates Metastatic cancer cell invasion and Adhesion. Sci Rep. 6:21564. doi: 10.1038/srep21564 26877098 PMC4753492

[CIT0018] Lampi MC, Reinhart-King CA. 2018 Targeting extracellular matrix stiffness to attenuate disease: from molecular mechanisms to clinical trials. Sci Transl Med. 10:422 eaao0475. doi: 10.1126/scitranslmed.aao0475 29298864

[CIT0019] Li J, Yuan J. 2008 Caspases in apoptosis and beyond. Oncogene. 27(48):6194. doi: 10.1038/onc.2008.297 18931687

[CIT0020] Li Y, Zhang Q, Tian R, Wang Q, Zhao JJ, Iglehart JD, Wang ZC, Richardson AL. 2011 Lysosomal transmembrane protein LAPTM4B promotes autophagy and tolerance to metabolic stress in cancer cells. Cancer Res. 71(24):7481–7489. doi: 10.1158/0008-5472.CAN-11-0940 22037872 PMC3261660

[CIT0021] Li H, Zhu H, Xu C-j, Yuan J. 1998 Cleavage of BID by caspase 8 mediates the mitochondrial damage in the Fas pathway of apoptosis. Cell. 94(4):491–501. doi: 10.1016/S0092-8674(00)81590-1 9727492

[CIT0022] Majeed F, Jadko S, Freedman MH, Dror Y. 2005 Mutation analysis of SBDS in pediatric acute myeloblastic leukemia. Pediatr Blood Cancer. 45(7):920–924. doi: 10.1002/pbc.20416 16007594

[CIT0023] Menne TF, Goyenechea B, Sánchez-Puig N, Wong CC, Tonkin LM, Ancliff PJ, Brost RL, Costanzo M, Boone C, Warren AJ. 2007 The Shwachman-Bodian-Diamond syndrome protein mediates translational activation of ribosomes in yeast. Nat Genet. 39(4):486. doi: 10.1038/ng1994 17353896

[CIT0024] Navab R, Strumpf D, To C, Pasko E, Kim KS, Park CJ, Hai J, Liu J, Jonkman J, Barczyk M, et al. 2016 Integrin alpha11beta1 regulates cancer stromal stiffness and promotes tumorigenicity and metastasis in non-small cell lung cancer. Oncogene. 35(15):1899–1908. doi: 10.1038/onc.2015.254 26148229 PMC4833874

[CIT0025] Nguyen TV, Sleiman M, Moriarty T, Herrick WG, Peyton SR. 2014 Sorafenib resistance and JNK signaling in carcinoma during extracellular matrix stiffening. Biomaterials. 35(22):5749–5759. doi: 10.1016/j.biomaterials.2014.03.058 24726537

[CIT0026] Ow Y-LP, Green DR, Hao Z, Mak TW. 2008 Cytochrome c: functions beyond respiration. Nat Rev Mol Cell Biol. 9(7):532. doi: 10.1038/nrm2434 18568041

[CIT0027] Provenzano PP, Inman DR, Eliceiri KW, Keely PJ. 2009 Matrix density-induced mechanoregulation of breast cell phenotype, signaling and gene expression through a FAK–ERK linkage. Oncogene. 28(49):4326. doi: 10.1038/onc.2009.299 19826415 PMC2795025

[CIT0028] Rice A, Cortes E, Lachowski D, Cheung B, Karim S, Morton J, Del Rio Hernandez A. 2017 Matrix stiffness induces epithelial–mesenchymal transition and promotes chemoresistance in pancreatic cancer cells. Oncogenesis. 6(7):e352. doi: 10.1038/oncsis.2017.54 28671675 PMC5541706

[CIT0029] Roos WP, Kaina B. 2013 DNA damage-induced cell death: from specific DNA lesions to the DNA damage response and apoptosis. Cancer Lett. 332(2):237–248. doi: 10.1016/j.canlet.2012.01.007 22261329

[CIT0030] Rujkijyanont P, Watanabe K-i, Ambekar C, Wang H, Schimmer A, Beyene J, Dror Y. 2008 . SBDS-deficient cells undergo accelerated apoptosis through the Fas-pathway. haematologica. 93(3):363–371. doi: 10.3324/haematol.11579 18268284

[CIT0031] Sharma S, Santiskulvong C, Rao J, Gimzewski JK, Dorigo O. 2014 The role of Rho GTPase in cell stiffness and cisplatin resistance in ovarian cancer cells. Integr Biol. 6(6):611–617. doi: 10.1039/C3IB40246K 24718685

[CIT0032] Visconti R, Grieco D. 2009 New insights on oxidative stress in cancer. Curr Opin Drug Discovery Dev. 12(2):240–245. 19333869

[CIT0033] Wajant H. 2002 The Fas signaling pathway: more than a paradigm. Science. 296(5573):1635–1636. doi: 10.1126/science.1071553 12040174

[CIT0034] Watanabe K-i, Ambekar C, Wang H, Ciccolini A, Schimmer AD, Dror Y. 2009 SBDS-deficiency results in specific hypersensitivity to Fas stimulation and accumulation of Fas at the plasma membrane. Apoptosis. 14(1):77–89. doi: 10.1007/s10495-008-0275-9 19009351

[CIT0035] Woloszynek JR, Rothbaum RJ, Rawls AS, Minx PJ, Wilson RK, Mason PJ, Bessler M, Link DC. 2004 Mutations of the SBDS gene are present in most patients with Shwachman-Diamond syndrome. Blood. 104(12):3588–3590. doi: 10.1182/blood-2004-04-1516 15284109

