# Supplementary figures and images for: Shwachman-Bodian-Diamond syndrome protein desensitizes breast cancer cells to apoptosis in stiff matrices by repressing the caspase 8-mediated pathway
Source: Anim Cells Syst (Seoul). 2019 Sep 20;23(6):414–21. doi: 10.1080/19768354.2019.1666030 (PMC6913620; doi:10.1080/19768354.2019.1666030)

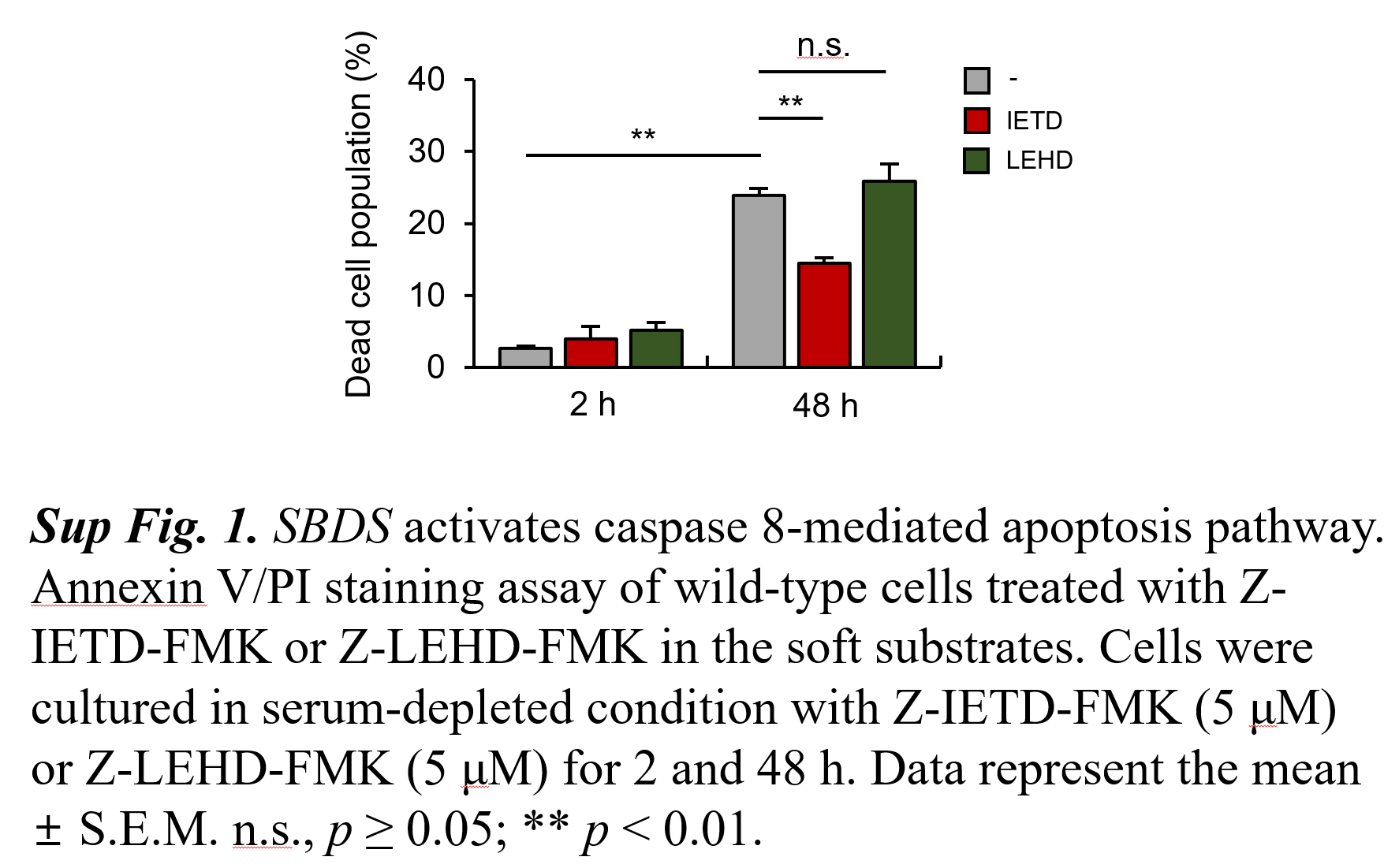

Supplement: Supplemental Material [file TACS_A_1666030_SM9317.tif]
